# Spectral measurement of daylights and surface properties of natural objects in Japan

**DOI:** 10.1364/OE.441063

**Published:** 2022-01-18

**Authors:** Takuma Morimoto, Cong Zhang, Kazuho Fukuda, Keiji Uchikawa

**Affiliations:** 1 Department of Experimental Psychology, University of Oxford, Oxford, UK; 2 Department of General Psychology, Justus-Liebig-Universität Gießen, Gießen, Germany; 3 Department of Information Processing, Tokyo Institute of Technology, Yokohama, Japan; 4 Department of Information Design, Kogakuin University, Tokyo, Japan; 5 Human Media Research Center, Kanagawa Institute of Technology, Atsugi, Japan

## Abstract

We present a spectral dataset of daylights and surface reflectances and transmittances of natural objects measured in Japan. Daylights were measured under the sun and under shadow from dawn to dusk on four different days to capture their temporal spectral transition. We separately measured daylight spectra at five different locations (including an open space and a forest) with minimum time difference to reveal whether a local environment alters daylight spectra reaching the ground. We found that colors of natural objects were spread in a limited area of color space, and data points were absent around saturated green regions. Daylight spectra were found to have a larger variation across time, weather, and local environments than previously thought. Datasets are made freely available, expanding past public datasets mainly collected in Northern America and Europe.

## 1 Introduction

The spectral shape of light reaching our eyes in everyday life is primarily determined by the spectral composition of daylights and the surface properties of objects that reflect and transmit the illuminant. Measuring spectral properties of daylights and natural objects has therefore been fundamental to vision research. However, a detailed characterisation is challenging partly because their spectral properties are highly mutable depending on various factors. For instance, types of natural objects such as flower, leaves, or fruits available at a certain location vary depending on climate, geology, or even eating habits. The self-rotation of the Earth creates a 24-hour cycle within a day which periodically alters color and intensity of daylights reaching the earth’s surface. Atmospheric conditions such as cloud cover, air pollution, haze or fog modify the spectral composition of daylight [1]. Furthermore, the tilt of the Earth’s self-rotation axis creates a variation in the length of the daylight period across seasons and geographical locations, suggesting that understanding daylight spectra requires comprehensive measurements in spectral, temporal, and spatial domains across diverse cities and countries.

Despite these challenges, past efforts have uncovered important statistical regularities in natural environments. Spectral reflectance functions have been measured and analyzed for a variety of natural objects including bark, flowers, fruits, grass, human skin and hair, leaves, lichen, pelage, plants, rocks, snow, soil, tree logs, and vegetation [2–14]. Such datasets have been invaluable resources which many researchers can use to address their own research questions. For example, a linear model defined by a relatively small number of parameters was shown to be effective in recovering the spectral reflectance of natural objects, leading to the development of heuristic-based color constancy algorithms under real-world situations [15,16].

Regarding daylight research, Judd et al. [17] performed a principal component analysis of 622 daylight spectra measured in three different countries (249 from Rochester, US; 274 from Enfield, England; 99 from Ottawa, Canada) and expressed a spectral composition of daylight using a linear combination of only three characteristic vectors. They also showed that most daylight chromaticities were aligned with a locus running near the Planckian locus. This locus is now recognized as a Commission internationale de l’éclairage (CIE) daylight locus, and the model has been widely used to synthesize typical daylight spectra that occur in natural environments especially in color science and color vision research domains.

The CIE daylight model is based on data measured in only three countries, and thus the true variability that exists around the world is presumably underestimated. However, the importance of incorporating geographical diversity into daylight measurements was already recognized in the 1960s, and this led to an international campaign to measure daylights, in which Australia [18], India [19–21], Japan [22] and South Africa [23] as well as Canada [24], United Kingdom [25–27], and United States [28] participated. These studies found a general agreement with the CIE daylight model. However, Hernandez et al. performed a large-scale measurement of 2,600 daylight spectra across two years in Granada, Spain [29], and reported larger spectral variations than the ones incorporated in the CIE daylight model. Moreover, it was shown that chromaticities of measured daylights are close to the CIE daylight locus, but they do not coincide particularly around the region of high correlated color temperature in color space. A more recent study extended measurements to twilight and night light in urban and rural areas in Philadelphia, USA, and found limited accountabilities of the CIE daylight model [30]. These observations imply that the estimate of variability in the natural lights remains inconclusive.

This study specifically considers two missing pieces in past measurements. First, the datasets collected in the international daylight campaign are generally not freely available, to our knowledge. Consequently, current publicly open datasets are still largely limited to narrow demographics, mainly Northern America (e.g., Spitschan et al’s dataset) and Europe (e.g., Hernandez et al.’s dataset). Second, because most past daylight studies were designed to precisely characterize the spectra of daylights reaching from the sun and sky to the earth’s surface, the measurements were generally taken place in a space widely open to the sky. However, daylights do not always directly reach our eyes in daily life. For example, local environments can consist of various objects, buildings, and in some cases trees. These obstacles in theory alter the spectra of daylight reaching the ground via reflection and transmission. For example, in environments covered by leaves (e.g., a forest), a spectrum of daylight is presumably largely modified by the transmittance of the leaves. Moreover, if we are outside on a sunny day, we see that many objects are in a casted shadow which direct sunlight does not reach, but the spectra of lights reflected from these shadow regions are little understood.

Thus, to further clarify the practical variability in spectral properties of objects and daylight in daily life, we measured new spectral datasets in Japan under a wider range of conditions in 2013 and 2014. We collected 359 natural objects in total and measured the reflectances of all objects (200 flowers, 113 leaves, 23 fruits, 6 vegetables, 8 barks, and 9 stones) and the transmittances of 75 leaves. To understand the practical variability of daylight we actually experience in everyday life, we measured daylights from dawn till dusk on four different days using a white calibration plate placed (i) under the direct sun to capture both sunlight and skylight and (ii) under a casted shadow to record skylight while blocking direct sunlight. We also separately measured daylights at five different locations (including a sports ground, a space between tall buildings, and a forest) with minimum time intervals to reveal the influence of surrounding environments on the spectral composition of daylights reaching the ground.

All measured data are made freely available in an external database. We expect that these datasets will be useful for several research fields that concern the spectral nature of natural lighting environments. First, the datasets allow us to model the variety of daily light exposure patterns in our retinae and especially their regularities which our visual systems may have evolved to exploit. Moreover, the importance of such spectral datasets was boosted by the discovery of intrinsically photosensitive ganglion cells which contribute to core biological functions, including the regulation of circadian rhythms [31]. Additionally, these datasets are also relevant to industry: for instance, display technologies might benefit from knowing the frequently encountered colors in the real world to optimize the color gamut of a display to cover natural color distributions. Artificial lighting could be designed to mimic the spectrum of daylights that can produce a naturalistic appearance of objects in the scene. For the industries of textile, pigment, or painting, data can be used to simulate and test the visual appearance of new products under different lighting environments.

## 2 Methods

### 2.1 Measuring spectral properties of natural objects

#### 2.1.1 Collection of natural objects

We collected 359 natural objects from the following categories: flower (200), leaf (113), fruit (23), vegetable (6), bark (8), and stone (9). Most objects were collected at Suzukakedai campus of Tokyo Institute of Technology (Yokohama, Japan). When we collected these samples, especially flowers, we took care so that colors of datasets included as diverse a set of colors as possible. We purchased some objects in the fruit and vegetable categories at a local grocery store. At the time of collection, we noted the scientific names of the flowers, leaves and barks from a name board provided by the University. If not available, we used a smartphone software which automatically identifies the scientific name from an input image (*PictureThis - Plant Identifier* developed by Glority Global Group Ltd.). The names of 2 flowers and 9 stones which could not be identified through either method were left blank in Dataset 1 [32] (‘datasheet’ hereafter).

#### 2.1.2 Spectral reflectance

**Fig. 1:**
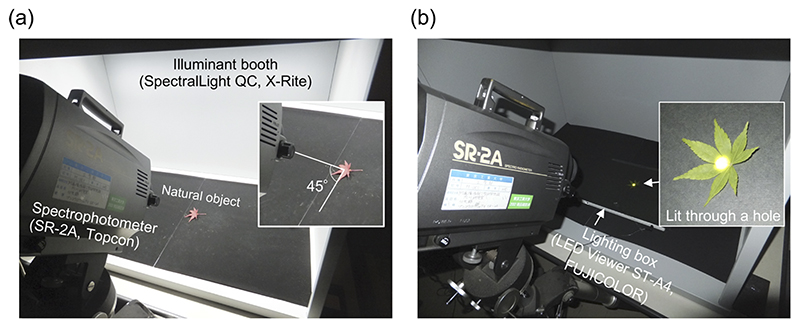
Setup to measure surface spectral properties of natural objects. (a) Measuring spectral reflectance. A natural object was placed in a light-booth that was set to emit a ‘D65’ light. The reflected light from a sample was measured using a spectrophotometer with an aperture of 1° of visual field from an elevation angle of 45° (380 - 780 nm, every 5 nm). The reflectance was obtained by dividing the spectrum of the reflected light by the spectrum of the illuminant itself separately measured using a white calibration plate with a flat reflectance. (b) Measurement of spectral transmittance. The sample was lit through a small hole on a lighting box, and the transmitted light was measured by a spectrophotometer. The spectrum of the transmitted light was divided by the spectrum of the light source to obtain the spectral transmittance.

We measured the surface spectral reflectance of 359 objects in the following way. As shown in Fig. 1, we placed a natural object in an illuminant-booth (SpectraLight QC, X-rite, Grand Rapids, USA) which equips a ceiling light covered with a diffuser to achieve a spatially uniform light distribution. We set the illuminant-booth to emit a ‘D65’ spectrum in order to illuminate the sample with a broad-band spectrum. We measured the spectrum of the light reflected from the sample from an elevation angle of 45° using a spectrophotometer (SR-2A, Topcon, Tokyo, Japan) with a 1° visual field aperture (corresponding to 123 mm on the object’s surface), from 380 nm to 780 nm with 5 nm steps. We separately measured the spectrum of ‘D65’ light *I* (λ) using a white calibration plate with a flat reflectance over visible wavelengths. We obtained the estimate of spectral reflectance *R*(λ) by dividing the reflected light from a sample *I* (λ)*R*(λ) by the measured illuminant spectrum *I* (λ). All measurements were performed in a dark room.

We found that some reflectance spectra physically shared too much of their reflectance. Thus, we identified reflectance pairs that have a Pearson’s correlation coefficient of more than 0.999 across 81 spectral channels (380 - 780 nm, 5 nm step) and removed one of the reflectances from each pair. The analysis of the remaining 307 natural objects is presented in the Results section in this paper, but the reflectance data for the excluded 52 samples are also included in the datasheet.

#### 2.1.3 Spectral transmittance

Panel (b) in Fig. 1 shows how we measured the spectral transmittances of 75 leaves. Each leaf was first flattened and then placed on a lighting box (LED Viewer ST-A4, FUJICOLOR, Tokyo, Japan) covered with black paper, and we measured the transmitted light that passed through the leaf by the spectrophotometer. We separately measured the spectrum of light emitted from the lighting box *E*(λ) and then a transmittance function *T* (λ) was estimated by dividing the transmitted light *E*(λ)*T* (λ) by *E*(λ). We measured the transmittance of each leaf both with the front side up (light entering from the back side of the leaf) and with the back side up (light entering the leaf from the front side). We conducted all measurements in a dark room.

### 2.2 Measuring daylight

#### 2.2.1 Measurement 1: Time-lapse of daylight spectrum on sunny vs. shadow regions

To characterize the temporal spectral change of daylight reaching a region under the sun and under a cast shadow throughout the day, we measured daylights from sunrise to sunset on 2013/11/20, 2013/12/24, 2014/07/03 and 2014/10/27. These four days were chosen as they have different durations of daylight hours. All measurements were performed on the rooftop space of a tall building (90.9 m from the ground) at Suzukakedai campus of Tokyo Institute of Technology (latitude 35.51° N and longitude 139.48° W) to avoid any obstacles that occlude daylight. The weather was mainly sunny on 2013/11/20 and 2013/12/24 and mostly cloudy on the other two days. The campus is located in an urban city near the capital of Japan, and thus measured daylight may include the influence of air pollution, for example the nitrogen dioxide which is known to absorb short wavelength light [1].

**Fig. 2:**
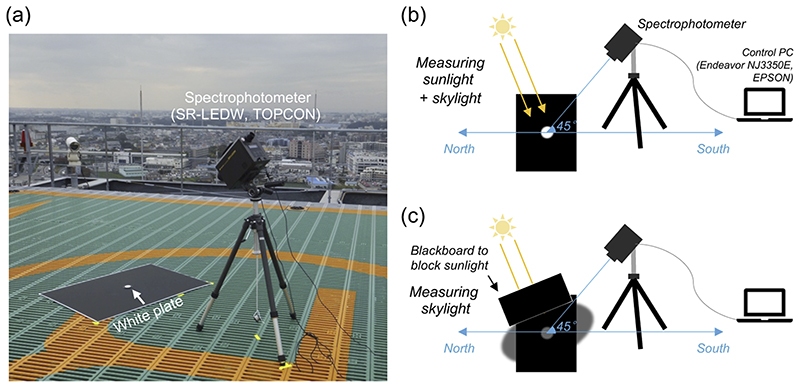
Measurement of spectral radiance of daylight. (a) Setup of measurement system. A white plate with a flat reflectance was placed on a black board to minimize the influence of inter-reflection from immediate surrounds. (b) Measurement of sunlight plus skylight radiance. A spectrophotometer was set at an elevation angle of 45°. The spectrum data from 380 nm to 780 nm with 1 nm step was saved to a connected control computer after each measurement. (b) The direct sunlight was blocked by the blackboard, and we measured the white plate under a casted shadow. This allowed us to measure mainly the skylight from other parts of the sky while minimizing the influence of direct sunlight.

A spectrophotometer (SR-LEDW, Topcon, Tokyo, Japan) was used to measure the spectral radiance of daylight spectra with wavelengths ranging from 380 nm to 780 nm with 1 nm step. The instrument was computer-controlled (Endeavor NJ3350E, EPSON, Suwa, Japan) and the measured data was saved after each measurement. As shown in panel (a) in Fig. 2, we placed a white calibration plate on a black paper to minimize any reflection from the immediate surround. Then, we measured the reflected light from the white calibration plate either under direct sunlight (panel (b) in Fig. 2) or under a casted shadow (panel (c) in Fig. 2). The first method allows us to measure both direct sunlight and skylight while the second method dominantly measures the skylight from other parts of the sky. In both methods, the instrument was set to focus on the white calibration plate from a 45° elevation angle. The aperture was set to 1-degree of visual field. We note that some early research (e.g. [29]) measured “hemisphere” daylight by orienting the photodetector of an instrument towards a zenith sky. Here we chose to measure the reflection light from a white plate using a spectrophotometer that has a narrow acceptance angle because (i) we aimed to measure lights that reach a reflecting surface in natural environments and (ii) it was easier to capture skylight reaching a casted shadow. We started the measurements before dawn. We obtained data every few minutes around sunrise and sunset as the light change was rapid around these periods, but otherwise the data was recorded approximately every hour. Measurements continued until the light level became too low to be reliably measured. The datasheet provides a visual estimate of the percentage of cloud cover across the sky at the time of each measurement. Most measurements were performed when the sun was not occluded by a cloud, but we also showed measurements performed when the sun was not visible. There was an aircraft warning lamp at the measurement site (seen around left middle region in panel (a) of Fig. 2), which turned on when it got dark. Since the lamp was not positioned near the measurement system, we expect that the influence on measured data is negligible, and thus no correction was applied to the data. Nevertheless, we noted in the datasheet when this lamp was on.

#### 2.2.2 Measurement 2: Daylight measured at different locations

**Fig. 3:**
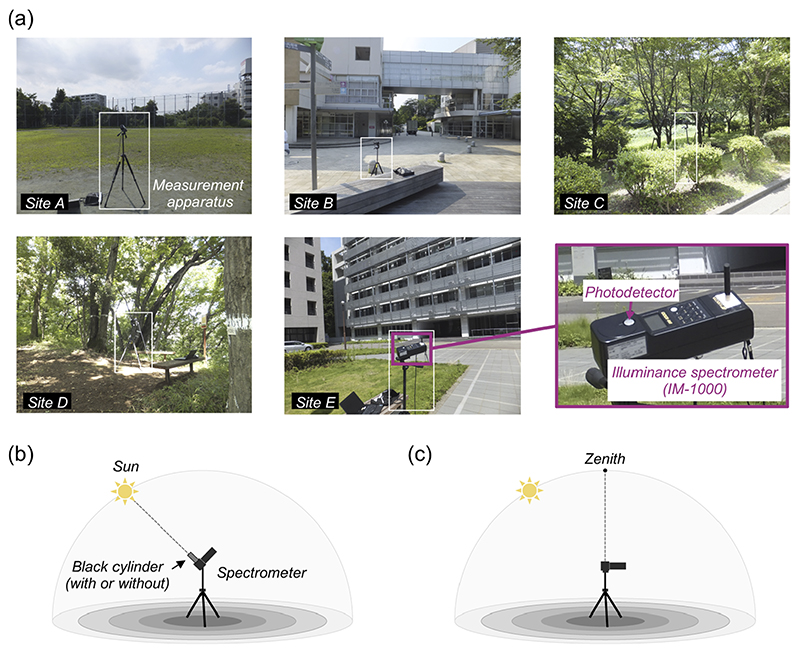
Measurement of spectral irradiance of daylights. (a) Five measurement sites: [A] a sports ground, [B] a wood deck near a school cafeteria building, [C] a space surrounded by trees, [D] a forest, [E] a space between tall buildings. (b) The measurement condition where the photodetector of the spectrometer was oriented towards the sun. The photodetector was covered (or not covered) by a black cylinder blocking light from other parts of the sky in order to increase the influence of direct sunlight. (c) Another measurement condition where the detector was oriented towards the zenith of the sky without a black cylinder aiming to capture hemispheric daylight including sunlight and skylight.

To investigate the influence of a local environment on the spectrum of daylight reaching the ground level, we measured spectra at five different sites within the University campus with minimum time gap on 2014/07/08. Figure 3(a) shows pictures of each location: [A] a sports ground, [B] a wood deck near a school cafeteria building, [C] a space surrounded by trees, [D] a forest and [E] a space between tall buildings. The density of trees and leaves was higher for site D than site C.

Only one measurement system was available, thus we conducted measurements at each site in order (A, B, C, D and E). It typically took around 10 mins to complete measurements at each site and move on to the next site; one measurement session completing all five sites took approximately 51 mins on average. This cycle was repeated twice in the morning (8:56 - 9:52 and 10:24 - 11:16) and 3 times in the afternoon (13:42 - 14:39, 15:00 - 15:43 and 17:07 - 17:54) on each day.

As highlighted by the white square in each picture of panel (a), we set an illuminance spectrometer (IM-1000, Topcon; 380 nm to 780 nm with 1 nm step) at each site at a height of approximately 120 cm. Then the photodetector of the instrument was oriented either towards the sun to maximize the influence of direct sunlight or towards the zenith of the sky to measure the light coming from the whole hemisphere (panels (b) and (c)). This measurement geometry is analogous to past studies (e.g. [18, 23, 29, 30]) except that we set up an instrument at a point of interest in a scene to capture the influence of local environments instead of a place open to the sky such as the rooftop of a building. When the detector was oriented towards the sun, we measured spectra in two ways: (i) one using a black cylinder covering the photodetector part to minimize the influence of light from other parts of the sky and (ii) the other without using a cylinder. We made sure that the detector was pointed towards the sun by minimizing the casted shadow of a black stick attached to the spectrometer (seen in bottom right picture in panel (a)).

Overall, the weather was partly cloudy on the measurement day. In the datasheet, we provided a visual estimate of the percentage of cloud cover at the time of each measurement. We attempted to take a measurement when the sun was not occluded by clouds, but it was not always possible. Thus, we also show measurements taken when the sun was covered by clouds in the datasheet.

## 3 Results

### 3.1 Spectral reflectance and transmittance of natural objects

**Fig. 4:**
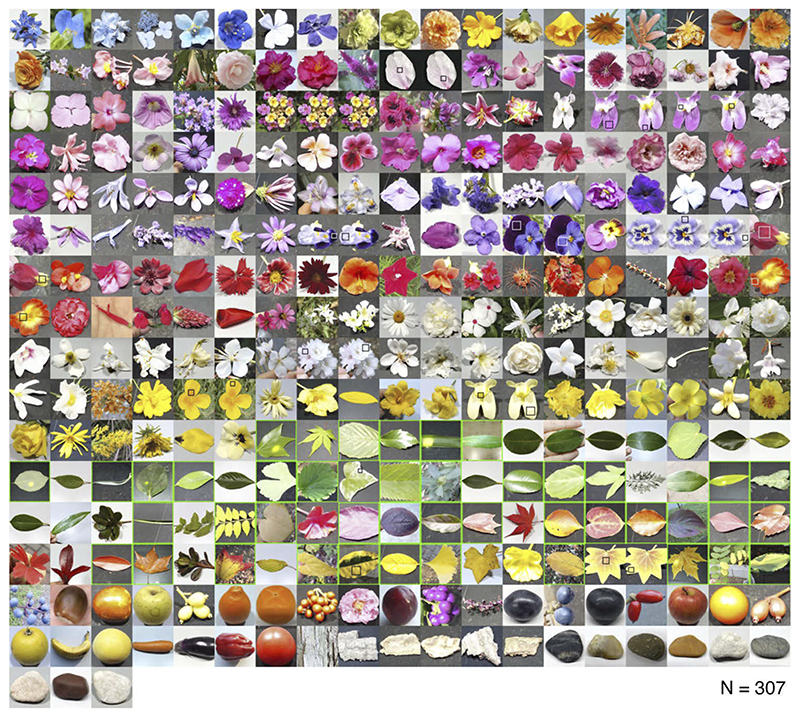
307 natural objects whose spectral reflectance was used for analysis. Black and white squares in each image (if included) approximately show the region at which the spectral reflectance was measured. Samples that are bordered by a green line show samples for which transmittance data was also measured.

Figure 4 shows the thumbnails of 307 natural objects whose reflectances were analyzed in this study. The photos are sorted by category (flower, leaf, fruit, vegetable, bark, and stone) and grouped approximately by the color of the object within the category. Leaves highlighted by a bright green square depict samples whose spectral transmittance was also measured. A black and white square in each photo, if included, denotes the approximate region at which the surface reflectance was measured.

**Fig. 5:**
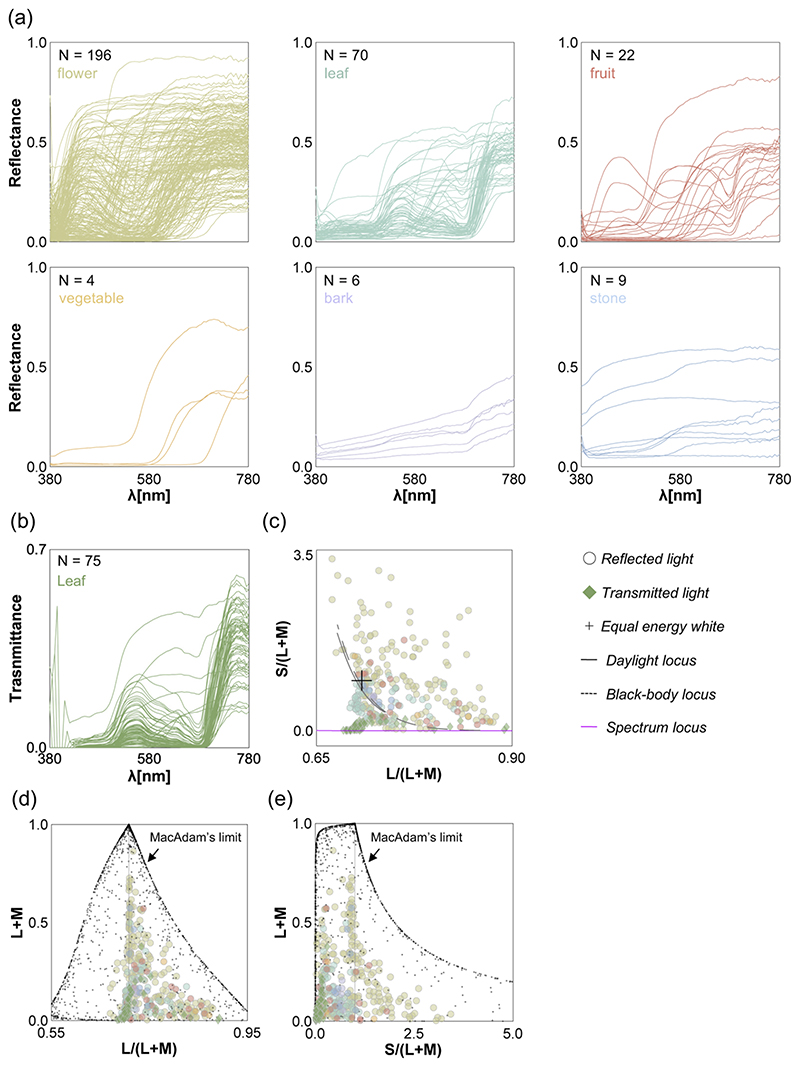
(a) Spectral reflectance for each object category shown at the left top corner in each subpanel along with the number of samples. (b) Spectral transmittance of 75 leaves. (c) Chromaticity distribution of reflectances and transmittances when placed under an equal energy white in MacLeod-Boynton chromaticity diagram. (d) L/(L + M) vs. luminance distribution. The vertical grey line shows the chromaticity of equal energy white. Each tiny black dot shows an optimal color, and distribution of all optimal colors (also known as MacAdam’s limit) shows the gamut of surface color. (e) S/(L + M) vs. luminance distribution.

Figure 5(a) shows the reflectance for each category. Objects in flower, leaf, fruit, vegetable, and bark categories generally have high reflectance around the long wavelength region while stones have flatter reflectances. Panel (b) shows the transmittances of the leaves measured with the front side up. The transmittances of most leaves have two peaks around middle wavelength and long wavelength regions showing a similarity to the reflectances of leaves. The transmittances for the other side of the leaves were consistent with this trend, and the data is provided in the datasheet.

We next calculated the chromaticity of the reflectances and transmittances when placed under an equal energy white light with a relative intensity of 1.0. Panel (c) of Fig. 5 shows the chromaticity distribution in the MacLeod-Boynton chromaticity diagram [33]. The horizontal axis is L/(L + M), expressing the ratio of signals in the long and middle-wavelength sensitive cones. The vertical axis denotes S/(L + M), expressing the signal of the short-wavelength cones in relation to a summed signals from long and middle-wavelength sensitive cones. The L/(L + M) and S/(L + M) of equal energy white denoted by a black cross symbol corresponds to 0.708 and 1.000, respectively. We used 2-degree Stockman & Sharpe cone fundamentals [34] to calculate the cone excitations. Overall, we see that chromaticities are spread across the chromaticity diagram. The transmitted light has notably low S/(L + M) values, coming close to the spectral locus plotted with a magenta line. The chromaticities of flowers are more widely spread, reflecting the wide variety of colors seen in the thumbnails of the flowers (Fig. 4).

Panels (d) and (e) of Fig. 5 show chromaticity vs. luminance distributions. Tiny black dots show optimal colors which have maximum luminances at the given chromaticities, and thus the distribution of optimal colors indicates a theoretically achievable gamut of surfaces colors under a particular illuminant [35]. The peak of the optimal color distribution corresponds to the chromaticity and luminance of a rendered illuminant (in this case the equal energy light with a relative intensity of 1.0). We see that compared to the theoretical gamut, the observed gamut for natural objects is substantially smaller. It is also notable that there are no samples at low L/(L + M) especially around high luminance region, meaning our measured data does not contain saturated greenish samples.

**Fig. 6:**
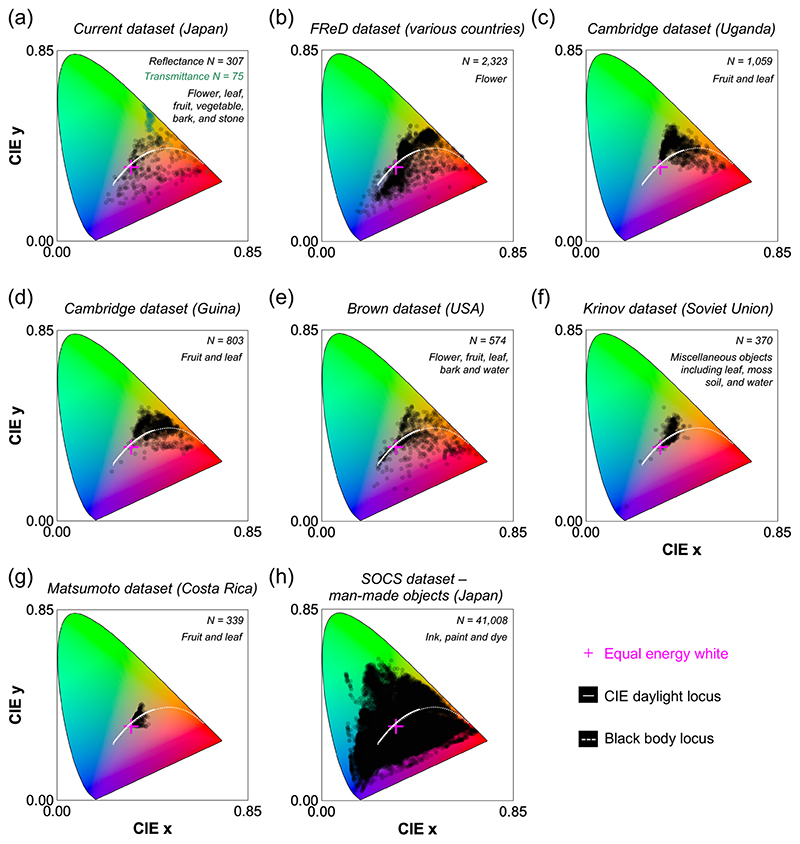
Chromatic distribution of various datasets for (a-g) natural objects and (h) man-made objects in CIE1931 *xy* chromaticity diagram. All chromaticities were calculated under equal energy white light. (a) Dataset collected in the present study. (b) FReD dataset including flower samples collected in diverse countries including Austria, Germany, Norway, Israel, and Brazil. (c, d) Cambridge dataset of fruit and leaf samples collected in Uganda and Guina. (e) Brown dataset including miscellaneous objects similar to our dataset. (g) Matsumoto dataset including fruit and leaf samples. (h) Man-made objects in SOCS dataset showing a substantially larger chromaticity gamut than the natural object gamut.

To see whether the lack of samples in a certain region of color space observed in Fig. 5 panel (c-e) is unique to our dataset, we compared the CIE1931 *xy* chromaticity gamut of our dataset to that of past reflectance datasets in Fig. 6. All chromaticities were calculated under equal energy white light. Panel (a) shows our dataset. Panels (b-g) show natural objects collected in different regions of the world (FReD dataset [4], Cambridge dataset [8,9], Brown dataset [10], Krinov dataset [11] and Matsumoto dataset [12]). Panel (h) shows man-made objects included in SOCS dataset (‘photo’, ‘graphic’, ‘printer’, and ‘paint’ categories) [36]. The FReD dataset includes reflectance samples collected in many different countries including Austria, Germany, Norway, Israel and Brazil. We also note that the original reflectances in the Krinov dataset lack data from 650 nm to 730 nm, and these missing values were linearly interpolated using reflectance values of neighboring wavelength bands. The chromaticity gamut of our dataset seems to be slightly smaller than FReD dataset especially around the blue region, but it is comparable with that of Brown dataset and larger than those of the Cambridge, Krinov, and Matsumoto datasets. The category of objects included in each dataset differs, but importantly all datasets of natural objects do not have samples around the saturated green region, meaning that this curious observation is not unique to Japan, but holds across different parts of the world. It is also notable that the gamut of man-made objects is substantially greater than that of natural objects. These analyses suggest that some colors, such as saturated green, may be only available from man-made materials, such as painting and ink, showing a systematic constraint in natural environments. However, we note that leaves collected in this study were relatively thick, and their transmittances were low. Thus, we would speculate that thinner leaves transmitting more light may create a more saturated greenish color.

### 3.2 Measurement 1: Time-lapse measurement of daylight throughout the day

**Fig. 7:**
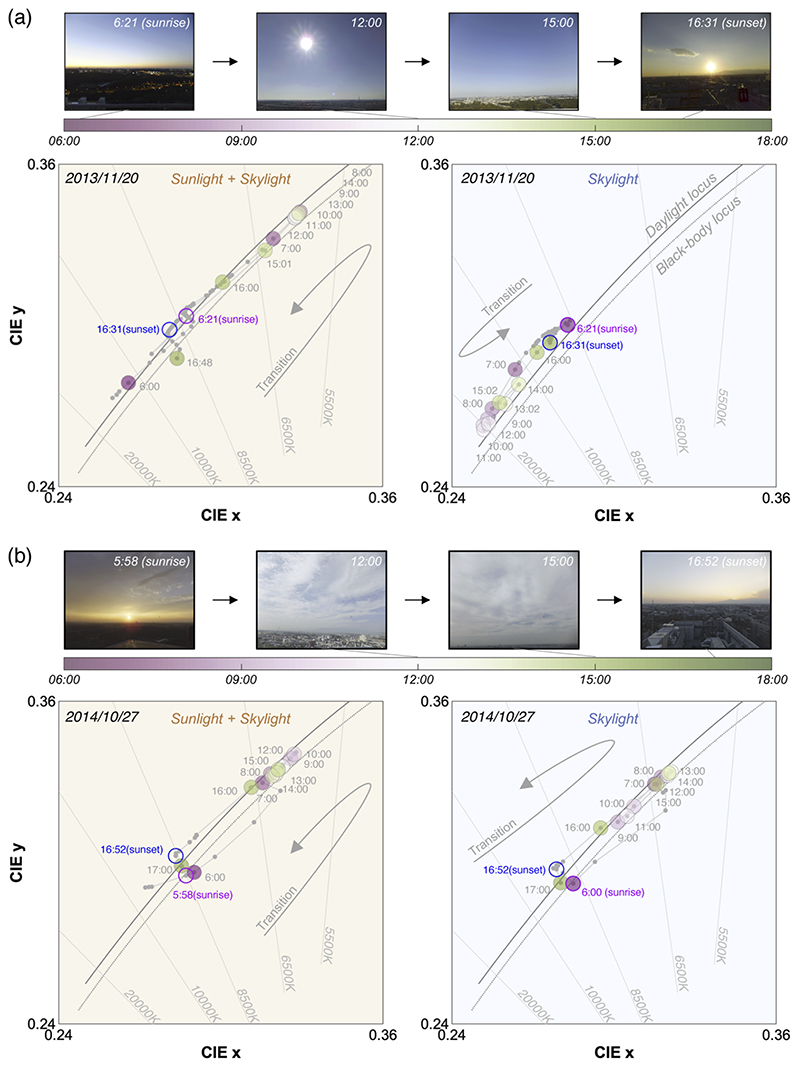
Temporal transition of daylight chromaticity in the CIE *xy* chromaticity diagram. (a) Measurement on 2013/11/20 (sunny day). Four pictures were included to show the climate condition on the day. The left subpanel shows measurement data taken without a blackboard and the right subpanel shows the data taken with a blackboard to block direct sunlight. We highlighted some data points with colored circles to denote measurements taken approximately from 6:00 to 17:00 every hour. We additionally circled some data points with magenta and blue to indicate data taken around sunrise and sunset. Small grey dots connected by a grey line show other data. (b) Measurement on 2014/10/27 (cloudy). Data format follows panel (a).

Figure 7(a) and (b) show chromaticities of daylight measured on 2013/11/20 (sunny) and 2014/10/27 (cloudy), respectively. Photos inserted at the upper part of each panel show the change in climate condition on the measurement days. Left subpanels depict the chromaticity of daylight spectra measured under direct sunlight, thus showing the chromaticity of the mixture of sunlight and skylight. Right subpanels show the chromaticity under a shadow region mainly reflecting the influence of skylight. Large colored circles (gradation between pink and green as shown in the color bar) depict data points measured at approximately every hour from 6:00 to 17:00. We additionally highlighted the data measured around sunrise and sunset with magenta and blue circles. Other small grey dots represent data points collected at other times. All dots were connected in temporal order to show the time-lapse of chromaticities.

The left subpanel in panel (a) shows that chromaticities change tightly along the CIE daylight locus (grey solid line) and blackbody locus (grey dotted line) throughout the day. At 6:00 am (before sunrise), the correlated color temperature (CCT) is more than 10000 K. From then, it rapidly decreases as the sun rises and increases back as the sun approaches sunset at 16:31. Curiously, the trend under shadow (the right subpanel) is the opposite of this. At 6:21, the CCT is around 8500K. However, it increases as the sun rises and decreases towards sunset. The CCT under the shadow region never became lower than 8500K on this day. These make sense as light hitting the casted shadow mostly comes from a blue sky on a sunny day. Overall, considering both subpanels, the CCT of daylight changes largely between approximately 6000K and > 20000K.

In contrast, on a cloudy day shown in panel (b), the transition of chromaticities behaves more similarly between sunlight-plus-skylight and skylight. This is presumably because the coverage of clouds makes the light distribution uniform across the sky. On this day, we did not observe any spectrum that had a CCT higher than 10000K, showing a substantially different range from the sunny day in panel (a).

We note that the results on the two other days were consistent with the results presented here. In other words, on 2013/12/24 (a sunny day) the transition of chromaticities between sunlight-plus-skylight and skylight was opposite to each other while on 2014/07/03 (a cloudy day) they moved in the same direction.

**Fig. 8:**
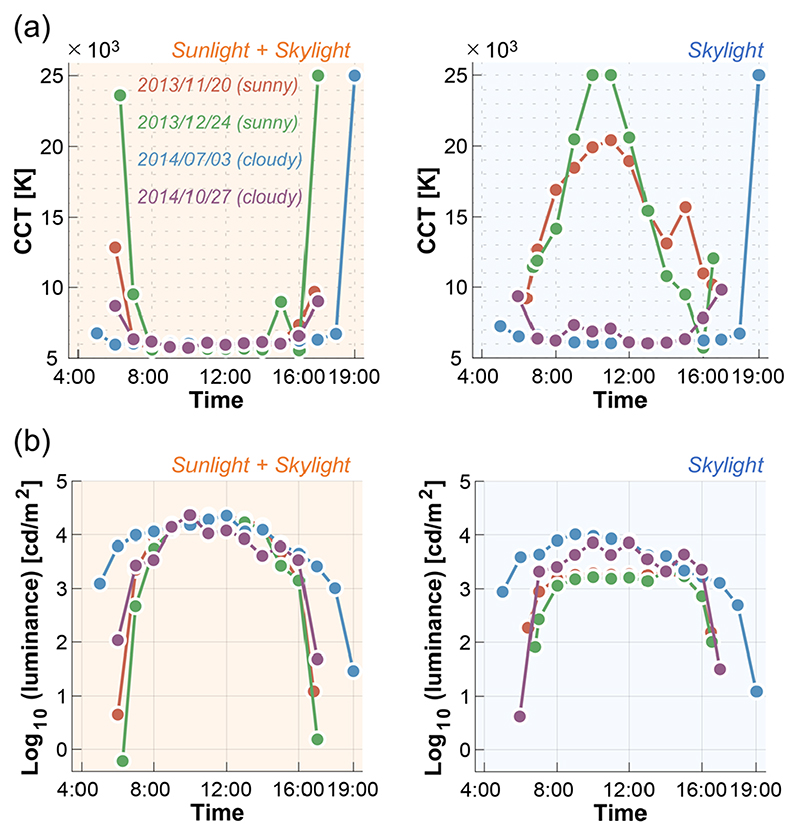
Transition of (a) correlated color temperature (CCT) and (b) log_10_ luminance as a function of time. Left subpanels show data for sunlight-plus-skylight and right subpanels show data for skylight.

Figure 8 summarizes the temporal transition of (a) CCT and (b) luminance for sunlight-plus-skylight (left subpanel) and skylight (right subpanel). As seen in the previous figure, the CCT for sunlight-plus-skylight shows a U-shaped transition on all measurement dates, while the CCT of skylight shows an inverted U-shaped change on sunny days and a U-shaped change on cloudy days. The luminance plots show an inverted U-shaped change which is similar between the two subpanels, but the luminance level of skylight is overall lower than that of sunlight-plus-skylight, as expected.

**Fig. 9:**
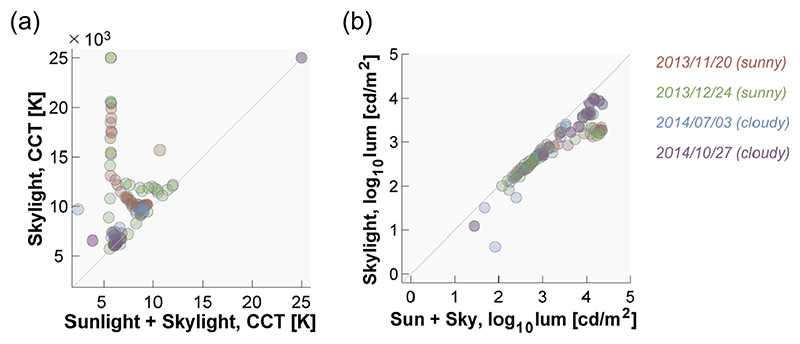
Scatter plot to show (a) CCT and (b) log_10_ luminance for total daylight (x-axis) and skylight (y-axis). Each data point has a pair of value for total daylight and skylight measured approximately at the same time. The color of the data points indicates the measurement date.

To evaluate the overall difference in CCT and luminance, we paired data points for total daylight (sunlight plus skylight) and for skylight that were measured at approximately the same time. Figure 9 shows 119 data points showing (a) CCT and (b) luminance of total daylight (horizontal axis) and skylight (vertical axis). The color of the data points indicates the measurement day. It is shown that skylight always has higher CCT and lower luminance than that of total daylight, as expected. One-tailed t-tests based on no assumption about equal variance confirmed that these trends are statistically significant (*t*(201.4) = 5.66, *p* = 5.19×10^−8^ for CCT; *t*(230.1) = 3.887, *p* = 6.64 × 10^−5^ for luminance).

### 3.3 Measurement 2: Daylight measured at different locations

**Fig. 10:**
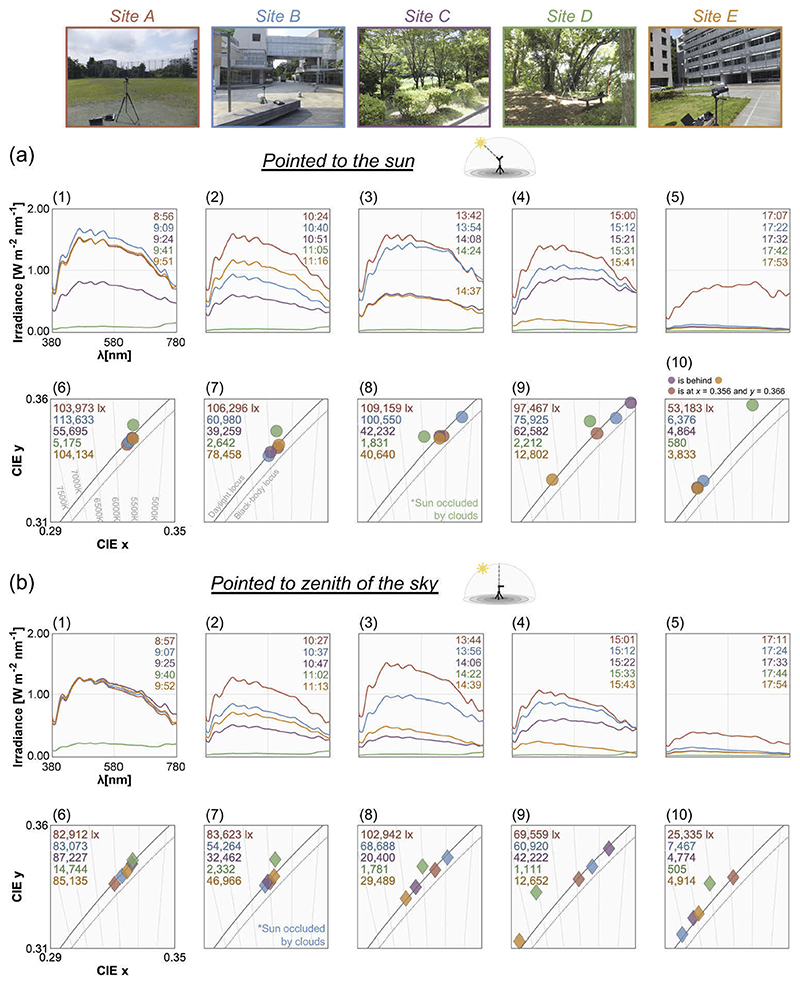
Measurement using an illuminance spectrometer at five different locations within the University. Sites A to E are coded in different colors. (a) Spectra and chromaticity measured by pointing a spectrometer shielded by a black cylinder towards the sun. Left two (labelled (1), (2), (6) and (7)) and right three subpanels (labelled (3), (4), (5), (8), (9) and (10)) depict data taken in the morning and afternoon, respectively. We show the exact time at which each spectrum was measured. Upper left numbers in the chromaticity plots show the illuminance value for each data point. (b) Spectra and chromaticities of half-hemisphere daylight measured with the photodetector pointing towards the zenith of the sky.

Figure 10 shows the irradiance spectra and chromaticities of daylights measured at five different sites. For data in panel (a), daylights were measured by pointing the photodetector of the spectrometer (shielded with a black cylinder) towards the sun while panel (b) depicts data measured by orienting the detector (without a black cylinder) towards the zenith of the sky.

First, as shown in panel (a) we found that spectral shape and intensity are largely influenced by the local surrounding environments. Throughout the day except for the fifth set of measurements (subpanel 5, 17:07-17:53), sites A and B generally have high intensity. The spectra measured in site D surrounded by dense trees shows a different trend. Its intensity is low, and the highest value of the spectrum is found at 780 nm. This is because the direct sunlight is spectrally filtered through the transmittance of leaves, thereby lowering the energy of sunlight and skylight especially around the short-wavelength region. The transmittance measured in this study also supports this view (panel (b), Fig. 5). Looking at the *xy* chromaticity diagram in panel (a), we see that for the morning measurements (subpanels 6 and 7), the CCTs of daylights measured at sites A, B, C and E are tightly aligned with the CIE daylight locus and are relatively stable across sites between approximately 5500K and 6000K, but it is notable that the light measured in site D systematically deviates from the daylight locus. The data from the afternoon measurements (subpanels 8, 9 and 10) also show a similar trend, but the color temperature spreads more widely across sites especially for the second and third set of afternoon measurements (subpanels 9 and 10). For the second afternoon measurement (subpanel 9), the orange circle shows the CCT around 6500K while others show lower color temperatures. If we look at the corresponding orange spectrum in the subpanel 4, we see that there is an overall decrease in intensity for this measurement. Though we confirmed that the sun was not hidden behind clouds when this data point was recorded, it is possible that an obstacle in the local environment might have partly blocked the direct sunlight, which consequently increased the relative influence of skylight, and the color temperature became relatively higher than other sites. For the third afternoon measurement (subpanel 5), the spectrum measured at site A shows much higher intensity than others. This is presumably because as the sun’s elevation angle gets smaller towards sunset, the direct sunlight is more likely to be blocked by obstacles in sites B - E. The CCT is also lowest for site A which again likely reflects a low elevation angle of the sun. Inserted values at the top left corner in each subpanel show the illuminance level, which largely varied across sites. The illuminance contrast between site A (overall highest illuminance) and site D (lowest illuminance) is about 51-fold (average across 5 measurements).

For panel (b), we see that intensity of the spectra is overall lower than the spectra in panel (a) as expected. If we look at the illuminance values in the chromaticity plots, we again see differences across sites, especially between non-forest sites and the forest. Furthermore, it is shown that overall chromaticities shift towards higher CCTs than those of panel (a). The second measurement in the morning (subpanel 7) shows a trend similar to the corresponding subpanels in panel (a) but this is because the position of the sun was close to the zenith of the sky around the measurement times. The deviation of chromaticities in site D from the CIE daylight locus is observed here, too.

In summary, the influence of local environments on the daylight spectra is evident. Although our intuition might be that daylights reaching the ground from sky are more or less similar regardless of the locations, results interestingly show somewhat large variations across measurement sites, implying that individuals receive largely different physical inputs to their visual system at different sites on the same day.

Note that the data measured with the photodetector pointed towards the sun without a black cylinder is not shown here but is provided in the datasheet. We also performed another set of measurements in the evening from 18:08 to 18:44 for sites A, B, C and D, but the measurement was aborted for site E as it became too dark to get reliable data. Thus, we did not include the analysis of this additional data in the main text, but they are provided in the datasheet.

### 3.4 Colorimetric and spectral analysis of whole dataset and comparison with CIE daylight model

Finally, we put together all the reflectances, transmittances and daylight spectra measured in the present study and performed summative colorimetric and spectral analyses. We also summarized the content of the dataset in Table 1 in the Appendix.

**Fig. 11:**
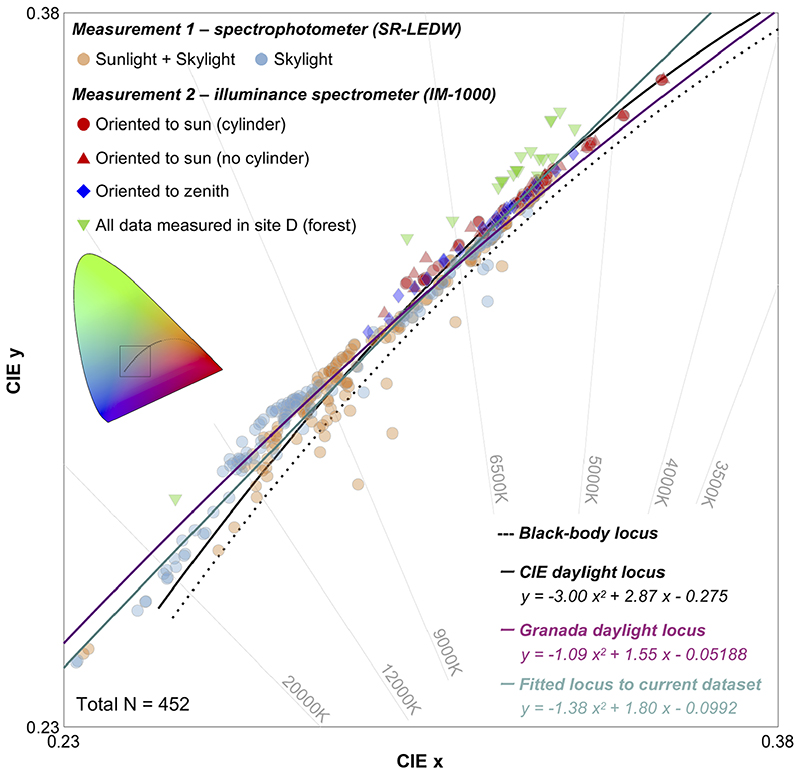
Summary chromaticity plot of 452 spectra measured in the present study. A black square region in the inserted colored ‘horseshoe’ diagram shows the region of the chromaticity diagram used in this figure.

First, in Fig. 11, chromaticities of all daylight spectra are plotted on the CIE1931 *xy* chromaticity diagram to see how chromaticities distribute in a color space mainly in relation to past daylight loci. Data points were fitted by a quadratic function using a least squares method. The resultant locus is shown with a cyan line. We excluded 25 data points which have less than 10 cd/m^2^ or 10 lx to avoid including data measured with low signal-to-noise ratio, and as a result there were 452 data points in total. For a comparison, the CIE daylight locus and Granada locus are shown with a black solid line and a magenta line, respectively. It seems that the locus fitted to the current dataset is generally close to the CIE daylight locus, but some data points clearly deviate around the high CCT region, in agreement with the Granada locus [28]. Data points with high CCT were mainly observed for time-lapse measurement under the shadow. Also, in measurement 2 where we oriented the photodetector to the zenith of the sky or the sun rather than measuring the reflected light from a white plate, we did not observe CCT higher than 9000K (except for one data point measured in the site D, forest environment). We speculate that this difference was generated mainly because that the area of the sky was larger for time-lapse measurement since it was performed on the rooftop and smaller for measurement 2 as local environments partially occluded the sky. Thus, the data points with high CCTs that deviate from the CIE daylight locus would be mainly explained by the dominance of skylight. It is also clear that spectra measured in a forest environment systematically deviate from the CIE daylight locus towards the greenish region in the color space. One might imagine that data measured in a forest environment should not be included in the estimate of the locus as a forest is not a standard environment to measure daylights. Thus, we performed fitting without data measured in a forest but the estimate of the locus remained approximately the same (*y* = −1.65*x* ^2^ + 1.94*x* – 0.119). This is because the proportion of forest spectra (21 out of 452 data points) are small and had little influence on the result of overall fitting. To summarize, our results generally show agreement with the widely used CIE daylight model, but disagreement around high color temperature regions and the influence of local environments on the effective daylight spectra should be noted.

**Fig. 12:**
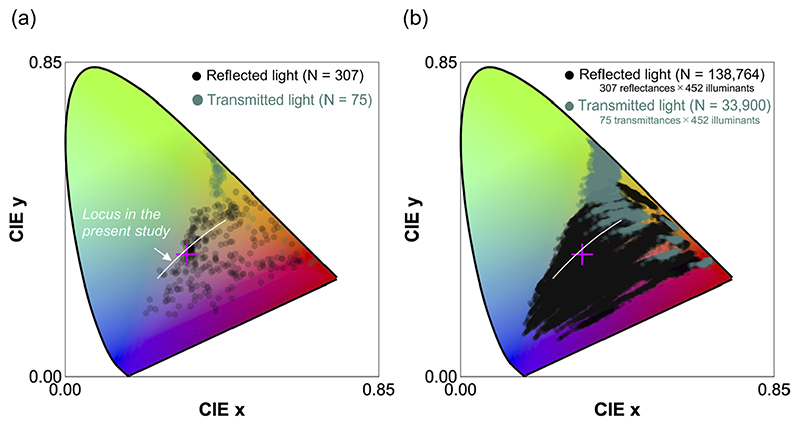
(a) Chromaticity distribution of 307 reflectances and 75 transmittances under equal energy white. The white line depicts a locus fitted to our dataset (cyan locus *y* = −1.38*x* ^2^+1.80*x*−0.0992 shown in Fig. 10). The magenta cross symbol shows the chromaticity of equal energy white. (b) Chromaticity distribution of 307 reflectances and 75 transmittances when placed under all 452 illuminants measured in the present study to see the range of natural color distribution. The number in parentheses shows the total number of data points. [two-column figure]

We next analyzed how much the color distribution of natural objects changes when we consider the spectral variation of daylights over time and across local environments measured in the present study. Figure 12(a) shows the chromaticities of 307 reflectances and 75 transmittances under an equal energy white (the same plot as panel (a) in Fig. 6), therefore showing the color distribution of natural objects under a single canonical illuminant. Panel (b) shows chromaticities of reflectances and transmittances when placed under all illuminants measured in this study. It is clear that the color distribution largely expands, yet a large proportion of color space is not filled with data points, implying that natural color distributions are somewhat limited, visualizing the strong constraint imposed to natural environments. It is also notable that some data points in transmitted light are close to the spectrum locus.

**Fig. 13:**
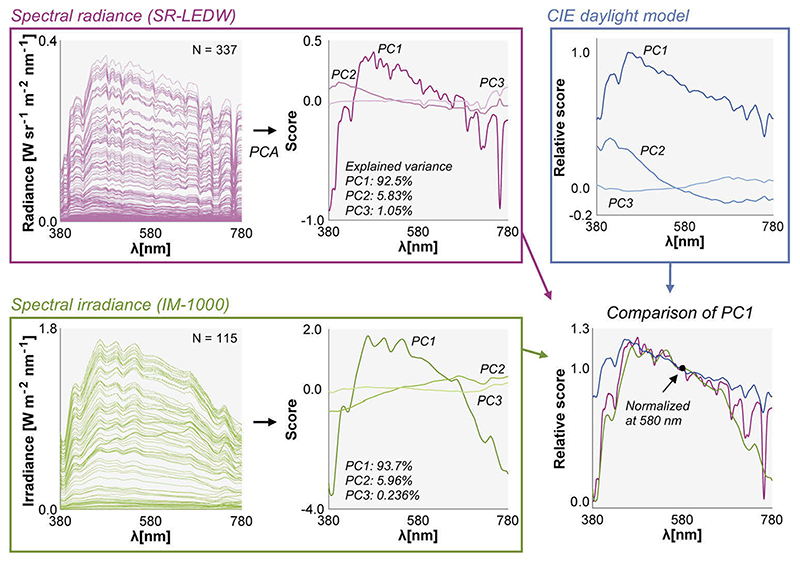
Spectral analysis of the dataset. We performed a principal component analysis (PCA) separately for 337 spectral radiance data and 115 spectral irradiance data. The first three characteristic vectors are shown in the middle graphs and the first principal components are compared with that of the CIE daylight model.

Finally, we performed a principal component analysis separately for radiance spectra (337) and irradiance spectra (115) as shown in Fig. 13. The results show that the first three components explain a large part of the observed variance in the datasets (> 99%) which agrees with the CIE daylight model. We also compared the first principal component explaining more than 90% of variance in each dataset in the right bottom subpanel. Each component was first scaled to have a minimum score of zero and then the spectra were normalized by the value at 580 nm. It was shown that the characteristic vectors found in the present study have a lower score at the short wavelength and long wavelength regions than that of the CIE daylight model.

## 4 Discussion

The purpose of the present study was to spectrally characterize the reflectance and transmittance of natural objects and daylights in Japan under a variety of conditions. The measured reflectance and transmittance dataset showed that the spread of chromaticities of natural objects in color space is somewhat limited, and data points were absent around the saturated bright green region. The daylight measurements showed that throughout the day the CCT of daylights largely varied between approximately 5000K and >20000K, and daylight spectra reflected from a white plate under direct sunlight and under casted shadow showed an opposite temporal transition on a sunny day. Furthermore, the chromaticity of daylights measured in a forest systematically deviated from the CIE daylight locus. These results revealed a novel variation in natural spectra which has not been previously characterized. One important message from the present finding is that true variation of natural daylight might be underestimated; more comprehensive measurements are needed to fully describe spectral and colorimetric variations in natural environments. The movement to measure real-world light exposure in daily life is active in research domains such as chronobiology, where the characterisation of “spectral diet” [37] plays a key role.

We believe that datasets describing natural scene statistics are becoming increasingly valuable for behavioural studies especially as a fair number of recent studies suggest that visual functions are potentially related to the regularities seen in natural environments (see review [38,39]). For example, the chromaticities of natural daylights were found to fall tightly on the locus which connects the chromaticities of 576 nm and 476 nm monochromatic lights that elicit unique yellow and unique blue percepts (see Fig. 10 [40]), respectively, suggesting a linkage between fundamental color percept and statistics in the real world. Another study showed that unique hues can change across seasons [41]. Furthermore, crucial color vision functions such as color contrast or assimilation are suggested to potentially occur by learning statistical regularities in external environment [42,43]. In the physiological domain, it was reported that the restriction of exposure to a variation of lightings could hamper the development of crucial visual functions such as color constancy [44]. Thus, measured datasets might provide insights into how and why specific visual functions are developed, addressing intrinsic questions in the field.

Furthermore, measuring datasets of natural colors in different cities and countries has an additional importance to understand universality and variability in physical visual inputs in different environments. However, publicly available daylight datasets to-date are mostly limited to specific regions in the world, namely Northern America and Europe. Especially if we are concerned about our visual functions at individual levels, such diverse datasets are vital in characterizing the statistics of local environments to which individuals living in different parts of the world are exposed. Our dataset helps mitigate a geographical bias in past datasets and promotes diversity and inclusion practices into vision research.

Our dataset was collected in 2013 and 2014, but it would be curious to question whether there has been a long-term change of the daylight spectra in Japan. One expected effect would be the change in the level of air pollution, especially nitrogen dioxide which is known to absorb short wavelength light [1]. Japanese industrial activities became particularly active during 1960s. Thus, the spectra around the time might look substantially different from the current datasets. There is no past daylight data in Japan that are publicly available, and thus the quantitative comparison would be difficult. However, many early daylight studies [17–27] were conducted in 1960s and 1970s, and thus measuring new datasets in the same countries now would allow us to characterize the long-term effect.

Finally, we here list a few limitations we recognize in our daylight dataset. First, due to the aim of the present study, we prioritized collecting data under a variety of condition, and thus the amount of data points we have measured is relatively small compared to past large-scale measurements (e.g. 2600 measurements in Hernandez et al. [29]). Second, time-lapse measurement was performed on limited days: sunny days in winter (2013/11/20, 2013/12/24) and cloudy days in summer and in autumn (2014/07/03, 2014/10/27). The purpose of this measurement was to investigate the temporal change of total daylight and skylight throughout a day and not to systematically characterize the influence of season or weather. Yet, systematic characterization of the influence of atmospheric conditions and seasonal variations might yield interesting insights. Furthermore, our daylight measurement relies on the current capabilities of spectrophotometers and an illuminance spectrometer, and thus datasets are still limited to understanding the summary statistics of lighting falling onto a single point. For example, recent studies performed directional spectral measurement using a mirror sphere and a hyperspectral camera which allowed characterization of lights coming from every direction towards a single point in a scene at once [45,46]. It is therefore important that future studies expand the present dataset. Nevertheless, even considering these limitations, we view this study as an important step towards understanding a practical variability of daylights across different times, local environments, and geographical locations. As more and more data collected in a diverse cities and countries become available in the public domain, it will also become possible to perform systematic analysis to shed a light on the geographical variations of natural lighting environments.

## 5 Conclusion

To understand the spectral properties of natural objects and daylights in Japan, we conducted a series of spectral measurements. Measured spectral reflectance and transmittance of natural objects showed a systematic constraint in a way that colors in the real world distribute in a color space. It was shown that the same constraint is observed in other datasets collected in different regions of the world. Time-lapse daylight measurements revealed a large temporal variation in chromaticity and intensity of daylights within a day, and the transition of correlated color temperature was different for regions under sunlight and casted shadow on a sunny day. Daylights measured at different sites uncovered that the spectrum of daylight that we consume in everyday life is potentially more variable than previously thought. Collected daylight data showed consistency with past datasets, but also showed some discrepancy, highlighting the importance of expanding measurements to wider conditions in order to understand the true variability in daylight spectra across countries and cities.

## Supplementary Material

Appendix

## Data Availability

All measured data in this study are freely accessible in Dataset 1 [32].
