# Supplementary material for: Spectral measurement of daylights and surface properties of natural objects in Japan
Source: Opt Express. Author manuscript; Available in PMC 2022 Jan 31. (PMC7612288; doi:10.1364/OE.441063)
Supplement: Supplementary file 1 [file EMS140850-supplement-Appendix.pdf]

Optics EXPRESS

Appendix

Table 1 shows the overview of Dataset 1 [32] measured in this study.

Funding. Japan Society for the Promotion of Science (26780413, JP17K04503, JP19K22881); Wellcome Trust (218657/Z/19/Z).
